# The prospective effects of workplace violence on physicians’ job satisfaction and turnover intentions: the buffering effect of job control

**DOI:** 10.1186/1472-6963-14-19

**Published:** 2014-01-17

**Authors:** Tarja Heponiemi, Anne Kouvonen, Marianna Virtanen, Jukka Vänskä, Marko Elovainio

**Affiliations:** 1 National Institute for Health and Welfare, P.O. Box 30, Helsinki 00271, Finland; 2 School of Sociology, Social Policy and Social Work, Queen’s University Belfast, Northern Ireland, UK; 3 UKCRC Centre of Excellence for Public Health (NI), Queen’s University Belfast, Belfast, UK; 4 Finnish Institute of Occupational Health, Helsinki, Finland; 5 Finnish Medical Association, Helsinki, Finland

**Keywords:** Job control, Work-related violence, Psychosocial resources, Intentions to quit, Physicians

## Abstract

**Background:**

Health care professionals, including physicians, are at high risk of encountering workplace violence. At the same time physician turnover is an increasing problem that threatens the functioning of the health care sector worldwide. The present study examined the prospective associations of work-related physical violence and bullying with physicians’ turnover intentions and job satisfaction. In addition, we tested whether job control would modify these associations.

**Methods:**

The present study was a 4-year longitudinal survey study, with data gathered in 2006 and 2010.The present sample included 1515 (61% women) Finnish physicians aged 25–63 years at baseline. Analyses of covariance (ANCOVA) were conducted while adjusting for gender, age, baseline levels, specialisation status, and employment sector.

**Results:**

The results of covariance analyses showed that physical violence led to increased physician turnover intentions and that both bullying and physical violence led to reduced physician job satisfaction even after adjustments. We also found that opportunities for job control were able to alleviate the increase in turnover intentions resulting from bullying.

**Conclusions:**

Our results suggest that workplace violence is an extensive problem in the health care sector and may lead to increased turnover and job dissatisfaction. Thus, health care organisations should approach this problem through different means, for example, by giving health care employees more opportunities to control their own work.

## Background

Health care professionals, including physicians, are at high risk of encountering workplace violence. For example, 59 per cent of Australian general practitioners reported that they had experienced work-related violence during the previous 12 months [1]. In US emergency departments, 75 per cent of physicians had encountered verbal violence and 28 per cent indicated that they had been victims of physical assault in the previous 12 months [2]. In another study, 96 per cent of physician respondents in US emergency departments reported experiencing verbal violence and 78 per cent a verbal threat during the previous 6 months [3]. In a study conducted among hospital and community physicians in Israel, 56 per cent reported verbal violence and 9 per cent physical assault during the previous year [4]. In Finland, every fifth physician reported having encountered physical violence or the threat of it in the previous year [5].

Workplace violence may have many negative ramifications for health care employees. Workplace violence has been associated with lower job satisfaction and higher levels of turnover intentions in nurses and home healthcare assistants [6,7]. Moreover, workplace violence has been found to affect negatively hospital personnel’s health [8] and increase sickness absences [9]. In physicians, work-related violence has been shown to lead to reduced job satisfaction and a decline in job performance [10]. In addition, among healthcare professionals, workplace violence may lead to difficulties in listening to patients, rumination, poor concentration, and intrusive thoughts [11], as well as impact negatively on family life and quality of life [4].

From the health care sector’s point of view, tackling workplace violence encountered by physicians is important given that it can lead to lower job satisfaction and increased turnover. Physician turnover is an increasing problem that threatens the functioning of the health care sector worldwide. Physician turnover may lead to decreased productivity, decreased quality of care and to an increased need to recruit and train new physicians. This is costly and may affect health outcomes [12,13]. In the US it has been estimated that the minimum cost of turnover may represent a loss of over 5 per cent of the total annual operating budget, due to hiring and training costs and productivity loss [14].

Job control refers to job and organisational characteristics, such as the employee’s decision-making authority, opportunities to participate, and opportunities to use skills and knowledge. Job control may have direct effects on job attitudes, health and wellbeing. In a study among Finnish anaesthesiologists, job control appeared as one of the most important work-related factors in relation to physicians’ work-related wellbeing [15]. Previous studies have repeatedly demonstrated the importance of job control for employees’ health. For example, low job control has been associated with increased myocardial infarction risk [16], increased heart disease risk [17], higher blood pressure [18], and to greater fibrinogen responses to stress [19]. Moreover, low job control has been associated with an increased number of sick-leave spells [20] and with poorer self-rated health eight years later [21]. In a study among emergency physicians, psychological health was not affected by the number and nature of hours worked but by the ability to control working hours and the perceived flexibility of the workplace [22].

High job control at work may protect employees from developing job dissatisfaction and psychiatric distress [23]. High job control may additionally increase organisational commitment [24] and decrease work-related anger [25]. A positive change in job control over a 4-year period was associated with higher levels of physical activity and self-rated health and lower levels of distress [26]. Job control has also been associated with job performance and ability to learn [27]. In addition, previous studies have shown that low control opportunities may affect employees’ attitudes to staying in or leaving a job, given that low job control has been associated with increased levels of retirement intentions [28,29]. In addition, job control has been found to mitigate retirement intentions associated with poor health and low work ability among physicians [30].

High job control may be viewed as a potential coping factor that helps distressed employees cope with demanding situations and, thus, lessen their job dissatisfaction and intentions to quit. According to Spector [31], job control can affect a person’s choice of coping strategy in a way that perceived high control is likely to lead to constructive coping, whereas lack of control is more likely to lead to destructive coping. Previous studies have indeed associated job control with successful coping [32,33] and successful coping, in turn, has been associated with fewer turnover intentions in demanding and stressful situations, such as with organisational change [34,35].

Job control may provide flexibility to avoid certain tasks that have a high risk of violence and to take breaks from work, which helps employees to regulate emotional responses and reappraise work challenges more positively [36]. Frese [37] has suggested that control enables a person to perform the most stressful tasks when that person feels particularly able to do them; that is, people can adjust the situation according to their needs, and can, therefore, be more relaxed in their work. Control may also act as a safety signal, given that a person with a high degree of control knows that he or she is able to change the situation if it becomes too difficult, thus knowing that the conditions may never be worse than he or she is willing to withstand [38]. Thus, many opportunities to control one’s job may act as a buffer against the negative effects of stressful working conditions like as work-related violence.

The aim of the present study was to examine the associations of work-related violence (physical violence and bullying) with turnover intentions and job satisfaction in a four-year follow-up among Finnish physicians. Specifically, we were interested to see whether job control would modify these associations. We hypothesised that both physical violence and bullying would be associated with increased levels of turnover intentions and decreased job satisfaction. We additionally hypothesised that job control would act as a buffer for these negative effects of work-related violence.

## Methods

**Figure 1:**
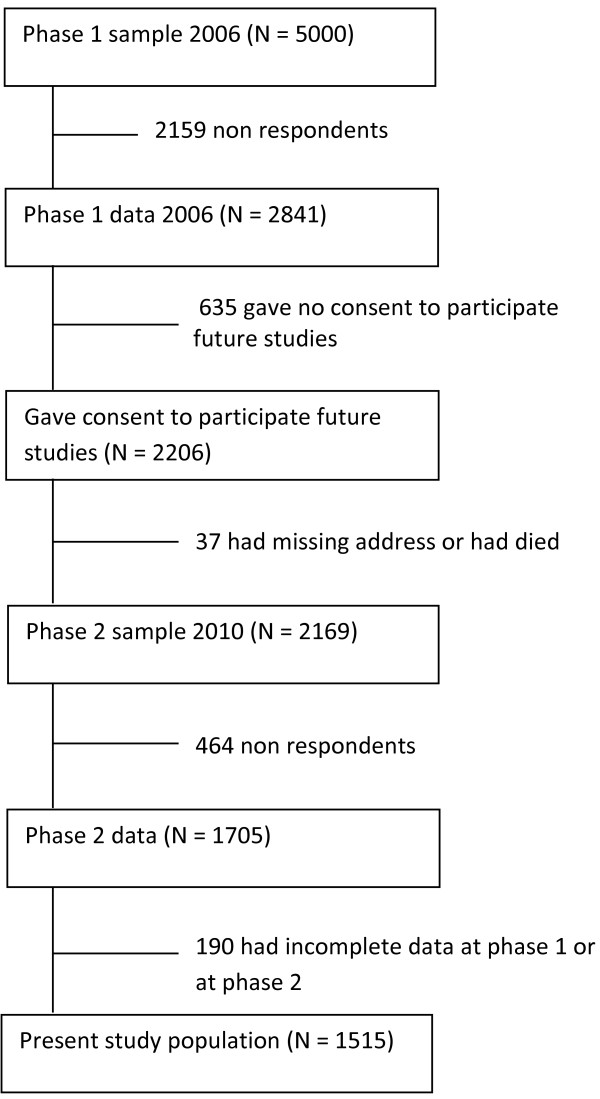
The study flow diagram.

The present study is part of the Finnish Health Care Professionals Study, in which we drew a random sample of 5000 physicians in Finland (30% of the whole physician population) from the 2006 database of physicians maintained by the Finnish Medical Association. The register covers all licensed physicians in Finland. Phase 1 data were gathered with postal questionnaires in 2006 (Figure 1). Non-respondents were twice sent a reminder and copy of the questionnaire. Responses were received from 2841 physicians (response rate 57%). The sample is representative of the eligible population in terms of age, gender, and employment sector [39].

Phase 2 took place four years later in 2010, with data gathered using either a web-based or a traditional postal survey. At phase 1 the respondents were asked their permission to participate in follow-up surveys and 2206 agreed. Those who had died or had incorrect address information were excluded (N = 37), thus, at phase 2 the survey was sent to 2169 physicians. First, an e-mail invitation to participate in the web-based survey was sent, followed by two reminders. For those who did not respond to these a postal questionnaire was then sent once. E-mail and postal addresses were obtained from the Finnish Medical Association. The total number of respondents was 1705 (response rate 79%), of which 1018 (60%) answered the web-based and 687 (40%) the postal questionnaire (the response format is adjusted for in the analyses). Of these, 190 had incomplete data and were excluded; the final study sample therefore includes 1515 physicians (61% women) aged 25–63 years (mean = 45.7 years) (2006). Ethical approval for this study was obtained from the Ethical Review Board of the National Institute for Health and Welfare.

### Measures

The present study used violence variables, job control, baseline turnover intentions, baseline job satisfaction, and covariates from phase 1. The outcome (turnover intentions and job satisfaction) variables were taken from phase 2.

*Job satisfaction* was assessed with the mean of 3 items derived from Hackman and Oldham’s [40] Job Diagnostic Survey on a 5-point scale, ranging from 1 (*totally disagree*) to 5 (*totally agree*). Cronbach’s alpha coefficient for this study was 0.66 at phase 1 and 0.88 at phase 2 (an example of the items: "I am generally satisfied with my work.")

*Turnover intentions* were measured with the mean of three questions concerning willingness to (a) change to other physician work, (b) to another profession, and (c) to quit (α = 0.61 at phase 1 and 0.66 at phase 2). The response alternatives were "1 = no, 2 = perhaps, and 3 = yes".

*Physical violence* was measured with a question asking whether the respondent had experienced work-related violence (such as kicking and hitting) or had been threatened with it and how often. Responses were coded as: 0 = never, 1 = less than once a year, 2 = once a year or more often. *Bullying* was asked with the following question: "Psychological violence means continuous repetitive bullying, victimising or offending treatment. Are you now or have you previously been a target of this kind of psychological violence and bullying in your own work?" The answer options were 0 = no and 1 = yes.

*Job control* was measured by combining the skill discretion (6 items) and decision authority (3 items) scales derived from Karasek’s Job Content Questionnaire JCQ [41]. Skill discretion measures how much the job requires skill, creativity, task variety, and learning of new skills (e.g., "My job requires that I learn new things"). Decision authority measures the freedom to make independent decisions and possibilities to choose how to perform work (e.g., "I have a lot of say about what happens in my job"). The items were rated on a 5-point Likert-scale, ranging from 1 (*totally disagree*) to 5 (*totally agree*) (α = 0.76 at phase 1).

Covariates included gender, age, specialisation status (specialist, specialisation on-going, and not specialised), and employment sector (hospital, primary care, and other).

### Statistical analyses

Analyses of covariance (ANCOVA) were conducted, with turnover intentions at phase 2 as the dependent variable, and physical violence, bullying, job control, gender, age, baseline turnover intentions, response format, specialisation status, and employment sector from phase 1 were included as independent variables. The analyses were conducted in four steps. In the first step, the univariate effects of physical violence, bullying, and job control for turnover intentions were examined in separate analyses (Model A). A second step included all the above-mentioned variables and gender, age, baseline turnover intentions, and response format (Model B). In the third step, specialisation status and employment sector were additionally added to the former model (Model C). Finally, the interactions of job control with physical violence and bullying were added (Model D). A similar series of ANCOVAs were generated with job satisfaction at phase 2 as for the dependent variable.

## Results

Table 1 shows the characteristics of the study sample. Sixty-one per cent had encountered physical violence in their career and 19 per cent had encountered bullying. The majority of the participants (77%) were specialised physicians and 46 per cent worked in hospitals, 22 per cent in primary care, and 32 per cent in other healthcare settings. The within-subjects differences in turnover intention and job satisfaction levels between phase 1 and phase 2 were examined with GLM repeated measures analyses. Analyses showed that job satisfaction levels had increased (F = 35.2, p < 0.001) and turnover intentions had decreased (F = 23.2, p < 0.001) during the study period.

**Table 1:** Characteristics of the study sample

	**n (%)**
Physical violence at phase 1	
No	596 (39)
Less than once a year	731 (48)
Once a year or more often	188 (13)
Bullying at phase 1	
No	1233 (81)
Yes	282 (19)
Gender	
Women	918 (61)
Men	597 (39)
Response format at phase 2	
Internet	922 (61)
Post	593 (39)
Specialisation status at phase 1	
Not specialised	166 (11)
Specialisation on-going	176 (12)
Specialised	1170 (77)
Employment sector at phase 1	
Hospital	694 (46)
Primary care	328 (22)
Other	493 (32)
	Mean (SD)
Age at phase 1	45.7 (9.5)
Turnover intentions at phase 1 (1-3)	1.54 (0.56)
Turnover intentions at phase 2 (1-3)	1.46 (0.52)
Job satisfaction at phase 1 (1-5)	3.96 (0.71)
Job satisfaction at phase 2 (1-5)	4.03 (0.83)
Job control at phase 1 (1-5)	3.95 (0.51)

Table 2 shows the results from the ANCOVA models for turnover intentions. Physical violence and bullying were associated with more turnover intentions, and job control was associated with less turnover intentions. However, only the association between physical violence and turnover intentions persisted after adjusting for baseline level, response format, demographics and work-related variables. Older respondents were less likely to have turnover intentions than their younger counterparts.

**Table 2:** The results of the analyses of covariance for turnover intentions

	**Model A**^**a**^		**Model B**^**b**^		**Model C**^**c**^		**Model D**^**d**^	
	**F**	**p**	**F**	**p**	**F**	**p**	**F**	**p**
Physical violence	19.8	<0.001	6.8	0.001	7.1	0.001	1.5	0.232
Bullying	11.9	<0.001	3.5	0.062	3.0	0.083	6.8	0.009
Job control	31.2	<0.001	0.3	0.584	0.1	0.735	1.9	0.166
Gender			0.2	0.682	0.2	0.608	0.5	0.498
Age			29.6	<0.001	33.1	<0.001	34.2	<0.001
Baseline turnover intentions			279.0	<0.001	278.4	<0.001	274.1	<0.001
Response format			3.5	0.063	3.7	0.056	3.3	0.069
Specialisation status					2.8	0.064	2.9	0.055
Employment sector					1.0	0.380	0.8	0.468
Physical violence*Job control							0.8	0.453
Bullying*Job control							5.7	0.017
R^2^				0.23		0.23		0.23

^a^Model A included univariate effects.

^b^Model B included physical violence, bullying, job control, gender, age, baseline turnover intentions, and response format.

^c^Model C included variables from model A and specialisation status and employment sector.

^d^Model D included in addition to Model C also interactions physical violence*job control and bullying*job control.

**Figure 2:**
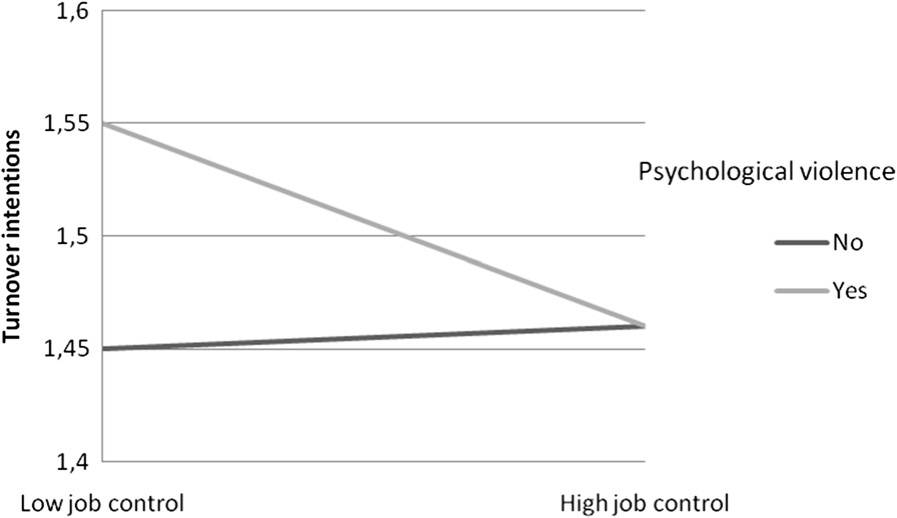
**The interaction between bullying and job control for turnover intentions.** Estimated marginal means among those scoring low (below median) and high (above median) in job control adjusted for baseline level, response format, demographics, and work-related variables.

The interaction between bullying and job control was significant for turnover intentions. As Figure 2 shows, job control was not related to increased turnover intentions among those who had not encountered bullying, whereas among those who had encountered bullying, job control was associated with turnover intention levels. That is, the highest levels of turnover intentions were among those who had low job control opportunities and had encountered bullying.

Table 3 shows the results from the ANCOVA models for job satisfaction. Physical violence and bullying were associated with lower levels of job satisfaction, and job control was associated with higher levels of job satisfaction. These associations persisted even after adjusting for baseline level, response format, demographics and work-related variables. Older respondents, those who answered by post, and those who worked in an employment sector other than hospitals or primary care were more likely to be satisfied with their jobs than their counterparts. The interactions with job control were not significant for job satisfaction.

**Table 3:** The results of the analyses of covariance for job satisfaction

	**Model A**^**a**^		**Model B**^**b**^		**Model C**^**c**^		**Model D**^**d**^	
	**F**	**p**	**F**	**p**	**F**	**p**	**F**	**p**
Physical violence	14.4	<0.001	5.6	0.004	4.8	0.009	0.2	0.799
Bullying	24.7	<0.001	10.2	0.001	9.9	0.002	2.4	0.123
Job control	81.2	<0.001	6.4	0.011	6.0	0.015	5.8	0.016
Gender			0.2	0.648	0.1	0.716	0.2	0.664
Age			6.5	0.011	9.6	0.002	9.8	0.002
Baseline job satisfaction			142.0	<0.001	136.0	<0.001	134.4	<0.001
Response format			5.4	0.020	5.3	0.022	5.1	0.024
Specialisation status					2.5	0.079	2.6	0.074
Employment sector					3.2	0.039	3.3	0.038
Physical violence* Job control							0.1	0.950
Bullying* Job control							1.2	0.265
R^2^				0.16		0.16		0.16

^a^Model A included univariate effects.

^b^Model B included physical violence, bullying, job control, gender, age, baseline job satisfaction, and response format.

^c^Model C included variables from model A and specialisation status and employment sector.

^d^Model D included in addition to Model C also interactions physical violence*job control and bullying*job control.

## Discussion

The present four-year longitudinal study showed that workplace physical violence and bullying were associated with decreased job satisfaction and increased turnover intentions among Finnish physicians. In addition, we found that opportunities to control one’s job were able to alleviate the increase in turnover intentions resulting from bullying.

Our results highlight the importance of job control as a buffer of negative psychosocial working environments. In addition, we found that job control was directly related to higher job satisfaction but the association between job control and turnover intentions did not remain significant after adjusting for baseline turnover intentions and demographics. Also previous studies have reported that job control is an important buffer. For example, high job control has been found to mitigate retirement intentions resulting from poor health and low work ability among Finnish physicians [30]. Furthermore, in a previous study high job control has been found to alleviate intentions to change profession that were associated with distress and sleeping problems [42]. Potential mechanisms behind this effect of job control could, for example, be that job control affects coping strategies, gives flexibility to avoid certain tasks and to take breaks to regulate emotional responses, gives possibilities to choose when to perform stressful tasks, and gives the assurance that a stressful situation can be changed if it gets intolerable [31,36-38].

Job control could be improved by giving employees a greater variety of tasks, opportunities to fully use and develop their skills, and a stronger voice in decisions. For example, participative decision-making has been introduced as a method to increase job control [43] along with greater freedom over start and finish times, more discretion over how tasks are performed, and autonomous or self-regulated work teams [44]. Young and Leese [45] have proposed greater flexibility as a potential solution for the problems in retention and recruitment of general practitioners. They suggested that flexibility could be improved by a) varying the time commitment across the working day and week (part-time, job-share, temporary, and short-term), b) offering wider choice of long-term career paths, c) offering more education and training, and d) widening the scope of remuneration and contract conditions. In a similar way, Shanafelt et al. [46] highlighted the importance of job autonomy as the central organisational characteristic that promotes well-being in physicians; they suggest that physicians should be provided with increased opportunity to influence their work environment, to participate in decisions, and to have more control over schedules.

Workplace violence is a big problem in health care and organisations should pay more attention to these issues. For example, in our study over sixty per cent of physicians had encountered physical violence in their career and approximately one in five had encountered bullying in the previous year. Targeting efforts at increasing control opportunities could alleviate the negative effects of workplace violence. Nevertheless, direct actions are also needed to actually decrease violence in workplaces. For example, metal detectors, security dog teams, cameras, and security personnel have been suggested to improve health care personnel’s security [47]. Hoag-Apel [48] suggested appointing a risk assessment team and staff training on, for example, body language, being alert to the tone of voice, and not taking anger personally. It has also been shown that reducing staff stress by improving staff’s cognitive efficiency and emotional control can lead to reduced violence [49].

In the present study, we found that physical violence led to increased turnover intentions and both bullying and physical violence led to reduced job satisfaction even after adjustments. Previous findings have associated physical violence, bullying, and verbal violence with both lower levels of job satisfaction and higher turnover intentions [6,7,10,50]. However, the previous findings were from cross-sectional studies, while our results here confirm that work-related violence also has longitudinal effects.

We found that older respondents were more satisfied with their jobs and were less likely to have turnover intentions than younger respondents. This corresponds well with previous findings among physicians [51-54]. In our study gender was unrelated to both job satisfaction and turnover intentions. Previous studies have found mixed results: Among German and Norwegian hospital physicians, gender was unrelated to job satisfaction [52], whereas among German general practitioners women had higher levels of job satisfaction than men [54]. In Chinese physicians, men had a higher likelihood of turnover intentions compared to women [53]. Moreover, we found that physicians working in hospitals and primary care were less satisfied than physicians from other sectors. This is congruent with a previous study showing that private sector physicians had higher levels of job satisfaction and organisational commitment and lower levels of psychological distress and sleeping problems compared to physicians working in the public sector [51].

The present study relied on self-reported measures, which may lead to problems associated with an inflation of the strengths of relationships and with the common method variance. In our study we were not able to differentiate between violence from patients or customers and violence from co-workers. Regarding workplace bullying the source is more likely to be co-workers than with physical violence. The effects of violence may vary depending on the source of violence, especially regarding bullying; that is, the effects of bullying might be different when caused by patients than when caused by co-workers. This issue should be investigated in future studies. Moreover, violence measures were collected 4 year prior to turnover intentions and we did not discriminate within the study population as to whether there were changes in violence experience over the course of the study frame. Therefore, it is possible that this may have caused a misclassification bias in our results. However, it is likely that this bias might simply weaken the associations found.

Moreover, although we controlled for age, gender, response format, specialisation status, and employment sector we cannot rule out the possibility of residual confounding. The present study used both a web-based and a more traditional postal survey to gather follow-up data. This is a limitation of the study. However, we controlled for this response format in the analyses. The use of web-based surveys is increasing, but they often result in low response rates, thus combining them with postal questionnaires might help to elevate response rates. There may be differences in response styles between the response formats and therefore it would be important to adjust for response format in analyses. Future studies about the subject are needed.

## Conclusions

Our results suggest that promoting employees’ control opportunities in health care organisations might help to provide a buffer against the negative effects of workplace violence on turnover intentions in physicians. In addition, we showed that physical violence and bullying has longitudinal effects on job satisfaction and turnover intentions. Workplace violence is an extensive problem especially in the health care sector and organisations should approach this problem through multiple means, such as, by giving health care employees more opportunities to control their own work, in addition to direct measures.

## Competing interests

All authors declare that they have no competing interest.

## Authors’ contributions

TH designed the study, directed its implementation, performed analyses and led all aspects of the work, including data analysis and writing. AK and ME contributed to planning the data analyses. AK, JV, MV and ME helped to conceptualise the ideas, interpret findings, and write and critically review drafts of the article. All authors read and approved the final manuscript.

## Pre-publication history

The pre-publication history for this paper can be accessed here:

http://www.biomedcentral.com/1472-6963/14/19/prepub
